# Conferences in the time of COVID: notes on organizing and delivering the first Brain Conference

**DOI:** 10.1093/braincomms/fcab142

**Published:** 2021-06-26

**Authors:** Lucia M Li, Niall J Bourke, Helen H L Lai, Hazel G May, Karl A Zimmerman, Joanne Bell, Eleanor Riches, Safa Abu-Sway, David J Sharp

**Affiliations:** 1 Division of Brain Sciences, Department of Medicine, Imperial College London, London W12 0NN, UK; 2 UK Dementia Research Institute Care Research and Technology Centre, Imperial College London and the University of Surrey, Sir Michael Uren Hub, London, W12 0BZ, UK; 3 Department of Bioengineering, Imperial College London, London, SW7 2AZ, UK; 4 Brain Editorial Office, Ormond House, London WC1N 3JZ, UK; 5 UKRI CDT Centre for Doctoral Training in AI for Healthcare, London SW7 2AZ, UK

**Keywords:** conference, online, *Brain* journal

## Abstract

To further fulfil their missions of promoting teaching, education and research in neurology and related clinical-academic disciplines, the Guarantors of Brain and the *Brain* journal family invited delegates to the first Brain Conference in Spring of this year. This event aimed to deliver excellent teaching and scientific presentations across a broad spectrum of neuroscience fields, with the key aim of making the content as accessible as possible. We hoped to capitalize on the benefits of an online format, whilst trying to capture a little of the joy of the in-person meeting. This article reports on the approach and practical choices made to achieve these goals, and we hope this will provide some guidance and advice to others organizing their own online conference.

## Teaser

Li et al. report on what went on ‘behind the scenes’ of the first Brain Conference, which took place in March 2021. Programming, technology and organizational choices are described, as well as reflections on what went well and what should be improved for future events.

## Introduction

As a charity whose aim is to ‘promote teaching, education and research in neurology and related clinical-academic disciplines’, the Guarantors of Brain has funded research fellowships, grants and educational activities since its inception. The latest venture, in conjunction with the *Brain* journal group, was an online conference which aimed to deliver excellent educational and scientific content to a wide audience. 

Here, we describe how an in-house team produced a 2-day programme for over 1400 delegates worldwide, which we hope will help others in planning similar events.

## Programme breadth and quality

It was important to produce a programme that would be interesting and educational for a broad neuroscience audience, clinical and scientific, in keeping with the missions of both the Guarantors of Brain and the *Brain* journal family. The programme was designed as a series of Topic Sessions encompassing the breadth of neuroscience topics within the publishing remit. The Guarantors of Brain and editors of *Brain* comprise neurologists and neuroscientists at the forefront of their field. A Chair for each Topic Session was approached, largely from this group, to curate each session. Each Chair was asked to invite speakers for a Teaching talk, a Basic Research talk and a Translational Research talk. They were asked to give consideration to diversity of location, career stage and demography. In this way, the substantial expertise and network of this group were leveraged to produce a line-up of highly distinguished speakers to deliver a programme of uniformly excellent talks, of both strong scientific and educational merit, across all topics (https://thebrainconference.co.uk/schedule/ Accessed 01 July 2021).

Promoting the work of early career researchers was an important aim of the programme, both as an innate goal but also as a means to showcase the newest research. To this end, Chairs selected two talks from submitted anonymized abstracts, which were embedded in each Topic Session. This ensured that those selected were of high quality, were consistent with the theme of the Topic Session, and would receive a substantial and interested audience. A number of the abstracts presented have since been published in journals, such as *Brain*, reflecting the quality of submissions received.

As the conference also coincided with a change of Editorship, passing from Dimitri Kullmann to Masud Husain, it felt fitting to have Alastair Compston give a keynote on ‘The History of *Brain*’. Alastair Compston closed the conference (with many of the audience enjoying the talk with a little *digestif* in hand) with a fascinating overview of how *Brain* has developed over time and, most recently, adding a sister journal in *Brain Communications*.

## Access

One of the most important aims was to broaden access as much as possible. To improve the international reach of the conference, the programme was split over 2 days, with an ‘early’ start on Day 1 (10 am GMT) and a ‘late’ start on Day 2 (1 pm GMT). In this way, we hoped to cover a wide selection of timezones to enable as many people to watch ‘live’ as possible. Additionally, a technical platform was selected which performed automatic browser-based recording of the talks. This meant that talks were available via the same link as the live conference, and accessible as soon as the live session was over, to anyone who had registered, rather like a streaming service. This availability continued for 2 weeks, which gave us time to edit and start uploading videos of individual talks to the *Brain* YouTube channel (https://www.youtube.com/channel/UClJJHH2xKQk8uZTC7-mJICg/featured Accessed 01 July 2021) for public availability. It is hoped that this channel will grow into a valuable library of information and education for future neurologists and neuroscientists.

Academic conferences are notorious for their expense, even without the added costs of national or international travel, which can be a barrier to attendance. The need for travel can also be exclusionary for those with caring responsibilities, or otherwise unable to travel. Enabling people to attend without leaving their own house is a happy side-effect of the online conferences. However, it was felt important to remove cost as a barrier to attendance. Each registration was priced at GBP10, which hopefully would have removed the cost barrier for the vast majority of people. There was also a voucher code to enable people to register for free. The hope was that this would particularly enable junior researchers, allied healthcare professionals and students, who may not have any study budget, to attend. This code was publicly listed on the registration website for use on an honesty system, and without people needing to provide evidence of financial hardship. Around 15% of attendees used this code.

An area of accessibility we hope to improve on for future conferences is that of providing live captioning in order to improve the experience for those who are hard of hearing, or for whom English is not the first language, for example, using browser-based plugins that provide live subtitling.

## Technology platforms

**Figure 1:**
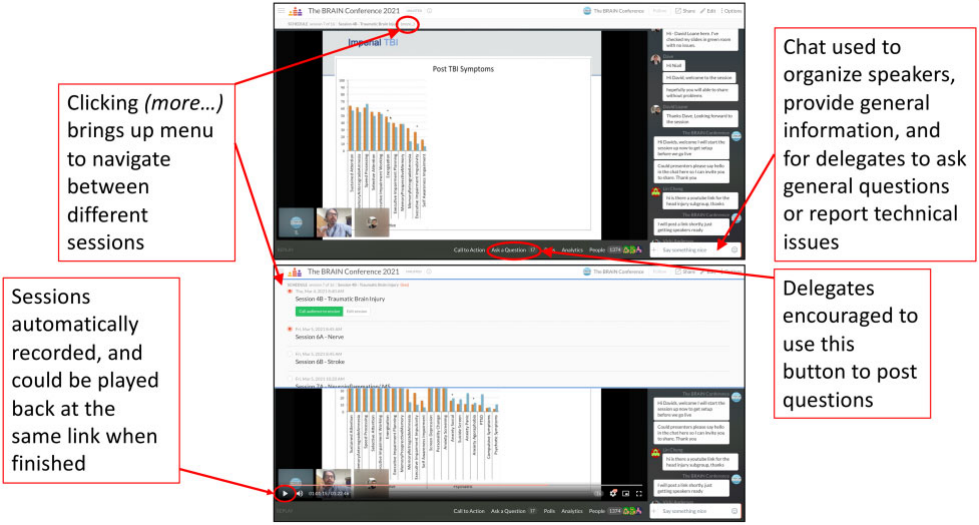
CrowdCast set up and features. Top panel shows the view for delegates, and bottom panel demonstrates navigation between sessions.

Technological platforms were chosen based on their ability to provide a smooth and engaging delegate experience. Crowdcast is a browser-based video conferencing platform that had a number of helpful features (Fig. 1). It permits parallel sessions that delegates can easily navigate between (Fig. 1), and also provides automatic recording of sessions. This was ideal for this conference given its wide geographical and subject scope. Delegates could enter any session they wanted to at any time, just like walking into different rooms at an in-person conference. If a session had already ended, the link took the delegate to an automatic replay of the session that they could watch in their own time, which meant that there was no wait for recordings to be uploaded. The web-based format meant that no downloads were required, as we were mindful that people may have been accessing the conference from phones or computers on which they did not have the admin rights to install programmes. The interactivity of CrowdCast is limited as delegates do not have video or microphone broadcast rights. However, the ‘Ask a Question’ function led to good engagement, with delegates being able to ‘upvote’ questions and speakers able to continue answering questions after their timeslot.

Due to the relative unfamiliarity of the platform, and also the fact that it functions poorly on browsers such as Safari, it was anticipated that some delegates would not be able to use CrowdCast. Therefore, the CrowdCast broadcast was streamed live to a private YouTube link that was provided at the start of the day.

The main disadvantage of CrowdCast was that it was not as well optimized across multiple platforms in the way, for example, that Zoom is, which has the potential to cause severe difficulties in allowing speaker video and sound, and screen-sharing. We attempted to mitigate this by inviting speakers to a tech run-through ahead of the conference and to send their slides ahead of time. Despite this, there were a few instances where even the most prepared speaker ran into technical issues, and the patience and flexibility of both audience and speakers were relied upon to do last-minute programme shuffles. Technical issues can of course occur during in-person conferences, but the physical separation of the speaker from the audience and IT support during online conferences does add an extra layer of stress to the proceedings. We advise all those organizing online events to pay particular attention to how technical support can be provided in the lead-up and on the day of the event.

**Figure 2:**
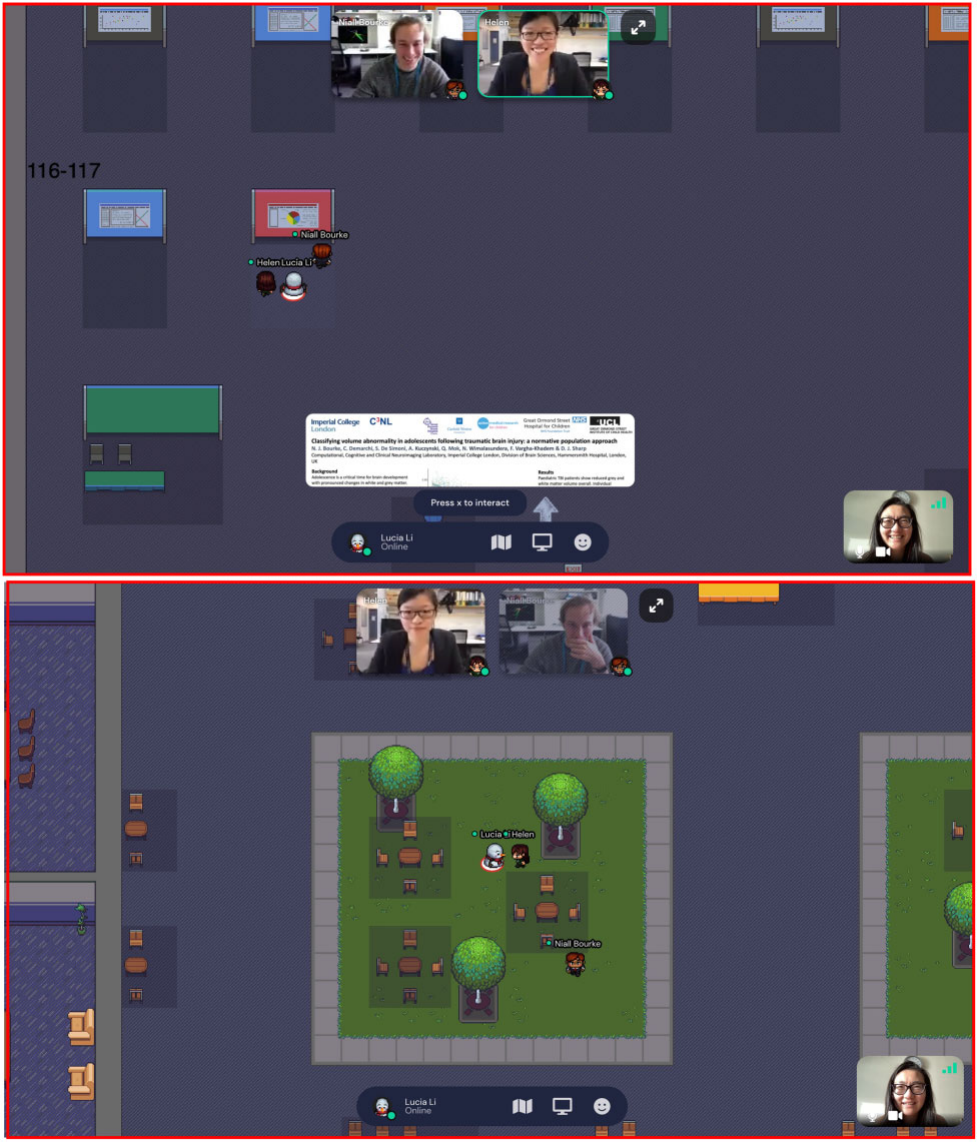
Poster session on Gathertown. Top panel shows how delegate avatars are able to interact with a poster and its presenter. Bottom panel shows how delegates’ cameras and mics are activated when close to each other (Helen and Lucia), but gradually fade away when moving further away from each other (Niall).

The poster session provided an opportunity to experiment with Gather Town, which aims to approximate the experience of in-person interaction. GatherTown enables the building of 2D environments that can be designed to simulate a conference hall, complete with space to ‘hang’ posters (Fig. 2). Delegates navigate around the space with an avatar (the happy default being a yellow-scarfed snowman) and can zoom in on posters they wish to see. A delegate’s video and mic are on at all times but only become visible and audible to other delegates when their avatars are physically near each other (Fig. 2). Delegates could also search and message other delegates directly. The conference feedback suggested that delegates enjoyed this format of seeing posters.

The other technological platforms used to deliver the conference included Microsoft CMT for abstract submission and Eventbrite for ticket sales. An advantage with both of these was that mass emails were possible, allowing for rapid communication with delegates.

## Conference organization and finances

The aim of the Guarantors of Brain was simply that enough people would pay for registration to cover the costs of running an online conference. This conference was organized entirely in-house, to avoid the high costs of engaging external conference providers. The core organization team was made up of seven clinicians and scientists, who received an honorarium, as well as a representative from *Brain*. This team managed the conference from start to finish alongside their ‘day jobs’, including: website and logo design, management of speakers, abstract submission, registration and running the conference on the day. Additional voluntary support on conference days was provided by five other university colleagues. Whilst the team already had recent experience of running an online conference, the ‘self-service’ nature of the technology platforms used was instrumental in enabling the in-house organization and delivery of the conference.

**Figure 3:**
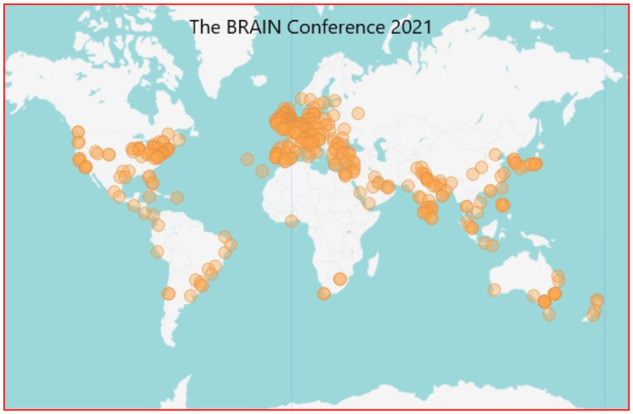
Heat map showing registrations by location around the world, with darker colour representing more people.

Our aim was to reach a global audience, both scientific and clinical. Therefore, online advertising formats were heavily relied upon. Promotion and advertising of the conference were largely online through campaigns on the *Brain* Twitter account, and through repeated advertising emails to national and international neuroscience and specialty organizations. Speakers were encouraged to advertise within their own networks in their own countries, and also leveraged some of the advertising capacity of *Brain*, e.g. through banners on the journals’ websites. The eventual audience was heavily skewed towards UK and European, with a decent number of North American and Australian delegates (Fig. 3). The aim for subsequent conferences is to continue to increase the geographical reach of the audience.

## Conclusions

While online conferences gathered momentum as necessary but less desirable replacements for in-person conferences in the COVID era, there are many advantages to this format. At their best, online conferences remove many of the traditional barriers to attendance, for both delegates and speakers, which can bring together a wider community. Innovative technologies are accessible and well-developed, allowing teams to deliver conferences for relatively low cost. Evolution of the conference format and delivery in a rapidly changing world is crucial to keep the advantages of online conferences, whilst reclaiming some of the joy of in-person meetings. The Brain Conference hopes to see you at future events, wherever that may be!

## Competing interests

D.J.S. is the Treasurer of the Guarantors of Brain. E.R. and J.B. work in the *Brain* editorial office. L.M.L., N.J.B., H.H.L.L., H.G.M., S.A.S., and K.A.Z. each received honoraria from the Guarantors of Brain for their role in managing the conference. 

